# Male Yawning Is More Contagious than Female Yawning among Chimpanzees (*Pan troglodytes*)

**DOI:** 10.1371/journal.pone.0040697

**Published:** 2012-07-11

**Authors:** Jorg J. M. Massen, Dorith A. Vermunt, Elisabeth H. M. Sterck

**Affiliations:** 1 Department of Behavioural Biology, Utrecht University, Utrecht, The Netherlands; 2 Department of Cognitive Biology, University of Vienna, Vienna, Austria; 3 Ethology Research, Biomedical Primate Research Centre, Rijswijk, The Netherlands; Max Planck Institute for Evolutionary Anthropology, Germany

## Abstract

Yawn contagion is not restricted to humans and has also been reported for several non-human animal species, including chimpanzees (*Pan troglodytes*). Contagious yawning may lead to synchronisation of behaviour. However, the function of contagious yawning is relatively understudied. In this study, we investigated the function of contagious yawning by focusing on two types of signal providers: close social associates and leaders. We provided a captive chimpanzee colony with videos of all individuals of their own group that were either yawning, or at rest. Consistent with other studies, we demonstrated that yawning is contagious for chimpanzees, yet we did not find any effect of relationship quality on yawn contagion. However, we show that yawn contagion is significantly higher when the video model is a yawning male than when the video model was a yawning female, and that this effect is most apparent among males. As males are dominant in chimpanzee societies, male signals may be more relevant to the rest of the group than female signals. Moreover, since chimpanzees form male-bonded societies, male signals are especially relevant for other males. Therefore, we suggest that the sex-differences of yawning contagion among chimpanzees reflect the function of yawning in the synchronisation of behaviour.

## Introduction

Recently, interest has grown in contagious yawning due to its proposed link with empathy [1]–[4]. Yawning contagion, where yawning in one individual enhances yawning frequency in observing individuals, or contagiousness of behaviour in general, is considered part of emotional contagion, the first and most basal level of empathy [1]; i.e. being affected by the emotional or arousal state of another individual [2]. Apart from theory, there is also empirical support that in humans contagious yawning is related to empathy, albeit indirect. The susceptibility to contagious yawning is positively correlated to questionnaire measures of empathy [3], and in a large multi-cultural data set the degree of yawn contagion could only be linked to the strength of the social bond [4]. Moreover, people with impairments in empathy (i.e. schizophrenics or people with autism spectrum disorder) appear to show impaired yawn contagion [3], [5]–[7]. From an ontogenetic perspective, however, children only develop yawn contagion after they have developed other empathy-based behaviours, even when the yawning model was their own mother [8].

Yawn contagion was first believed to occur only in humans (*Homo sapiens*) [9], but has now also been reported in chimpanzees (*Pan troglodytes*) [10], [11] and gelada baboons (*Theropithecus gelada*) [12]. Similar findings were reported for stump-tail macaques (*Macaca arctoides*) [13], yet it remains unclear whether these resulted from actual yawn contagion or were driven by tension [13]. In addition, two studies also reported contagious yawning in domestic dogs (*Canis familiaris*) [14], [15], yet two other studies failed to replicate these results [16], [17]. Furthermore, three recent studies in non-human animals provided indirect evidence for a potential link between yawn contagion and empathy [12], [15], [18]. Empathy is related to the relationship with an individual, and is expected to be more apparent when individuals are socially closer, more familiar or more similar [19]–[21]. In line with this argument, a study on gelada baboons demonstrated a positive correlation between the contagiousness of yawning and grooming levels between dyads; i.e. individuals that were socially closer appeared to be more prone to contagion of each other’s yawns [12]. Similarly, a recent study on chimpanzees demonstrated an ingroup-outgroup bias for yawn contagion; i.e. individuals yawned more in response to a yawning individual from their own group than in response to yawning strangers [18]. Finally, a study on keeper-dog contagious yawning found that the yawning of a familiar caretaker was more contagious to dogs than the yawns of unfamiliar humans [15]. Although indirect, these results strengthen the idea that also in non-human animals a link exists between contagious yawning and empathy. Nevertheless, empathy may operate as a (proximate) mechanism affecting the susceptibility to yawning contagion, yet does not provide an evolutionary (ultimate) function of this behavior. Consequently, the function of yawn contagion was not investigated in these studies and remains relatively understudied.

From a functional (ultimate) perspective, it has been suggested that yawn contagion causes synchronization of group behaviour, by synchronization of rest-activity transitions [22]. Emotional contagion, suggested to be present in chimpanzees [23], may facilitate this process, as it causes adoption of another’s emotional or arousal state [22] and may in turn result in better behavioural synchronisation with preferred animals [cf. 12], group members [cf. 18], or caretakers [cf. 15].

Here, we investigated the function of yawn contagion and its proposed link to empathy in a captive group of chimpanzees. We hypothesised that if contagious yawning has a communicative function, yawn contagion will be higher when the relevance of the signal is high; i.e. when provided by a socially close individual and by those individuals that decide upon group movement. This results in two predictions. First, we expect that yawn contagion is more prevalent among individuals with a good than a bad quality relationship. Second, as in chimpanzees males are dominant [24] and guide the movements of their groups [25], [26], we predict that yawn contagion will be higher if a male provides the signal. We presented 15 adult chimpanzees yawn and control videos of all group-members and measured their response (i.e. yawning). Thereafter, we correlated the contagiousness of yawning with the relationship quality with the target individual. Moreover, we compared the contagiousness of yawns of the different sexes.

## Materials and Methods

### Ethics

All testing was conducted as a part of the chimpanzee enrichment program of Burgers’ Zoo (Arnhem, the Netherlands). The research activities were fully integrated into the daily routine and required no manipulation of individuals. In fact, the chimpanzees were basically only observed in their home enclosure, yet after presenting them with video-projection on a wall of their enclosure. Consequently, the chimpanzees were never deprived of water and food at any stage. Therefore, this study was conducted in compliance with all relevant Dutch laws and in agreement with international and scientific standards and guidelines. Furthermore, due to the non-invasive character of the study and absence of any potential discomfort, our study did not meet the definition of an animal experiment as mentioned in Article 1 of the Dutch ‘Experiments on Animals Act’. Consequently, the ethics committee of Utrecht University waived the need for approval.

### Subjects

Fifteen chimpanzees were tested; 3 adult males and 12 adult females, which were all housed in one social group in Burgers’ Zoo, Arnhem, the Netherlands. The group was established in 1971 and most individuals were born in this group. The group is housed in an indoor enclosure (21 × 18 m) with access to a very large outdoor compound (0,7 ha), both furnished extensive enrichment devices. The group was fed a diet of fruit, vegetables, and commercially available monkey chow. Water was available ad libitum.

### Apparatus

A spontaneous, full (i.e. non-tension) [13], [27] yawn from each of the 15 individuals was recorded with a digital camcorder (JVC, Everio S, GZ-MS215). Each yawn was edited to a 14 s clip (using Windows Live movie maker) and slightly slow-motioned (0.75×) to assure that the stimulus length was sufficiently long to be detected by the group. For the control video, we selected a 14 s segment from the same footage from the same individual shortly before or after the yawn (also 0.75× slow-motioned). The yawn or control clips were repeated four times in each video and separated each by one second of red screen. Prior to the clips and red screens, a 30 s primer of one of three caretakers was added to attract the attention of the chimpanzees. Consequently, the order of clips on the videos was: primer – red screen – yawn/control clip – red screen – yawn/control clip – red screen – yawn/control clip – red screen – yawn/control clip, with a stimilus duration of 60 s and a total duration of 90 s.

### Procedure

The chimpanzees were tested in their indoor enclosure as a group. Per day, we presented the group with four videos that were projected 2 m high on the wall by a projector (Epson EB-1723, LCD, projection area of circa 100×100 cm). The order of projected individuals was randomized, but the yawn and control videos of one individual were always presented together in one session (in balanced order), to increase the chance that the same subjects were watching. After the first video (yawn or control) was presented, a 3.5-minute observation period followed. Then the second video was projected (when the first video was a yawn video, the second was a control of the same subject and vice versa), again followed by a 3.5-minute observation period and so on. All videos were shown twice to the group with the order of yawn and control video counterbalanced per projected individual. Two digital camcorders (JVC Everio) recorded all individuals during the test sessions from two different angles and data were, thereafter, coded double blind by DAV. We measured per individual: attention towards the projection (1/0) during the time (60 s) that the yawns/non-yawn videos were shown to them, and the number of yawns given. Individuals that did not pay any attention to a video were excluded from the analyses of the video’s model.

We assessed grooming relations and proximity patterns by conducting time-sample scan observations of all group members four times a day (2–4 days a week) during a period of eight months. These observations were conducted randomly throughout the day, yet had to be at least one hour apart to ensure independence of the data [28]. From these data, a continuous measure of relationship quality measured as grooming frequency per dyad was calculated [cf. 12].

### Data Analyses

To avoid an effect of the fact that yawning often occurs in bouts (i.e. yawns often come in series, so a first yawn may cause an individual to show additional yawns), we used the proportion of videos to which an individual responded with at least one yawn as a measure for further analyses. In addition, per projection we did not use the yawning data of the individual that was watching its own projection. Finally, in our response data we encountered one case in which we noticed a series of yawns of individuals that were sitting close to each other. We omitted these data from our analyses, since we could not establish whether these yawns reflect the contagiousness of the yawn/control video or that of the conspecific sitting in close proximity. We cannot fully exclude this explanation for the rest of our data. However, in general the chimpanzees were sitting rather spread out over the enclosure and the yawns we measured were also rather spread out over time.

We compared proportions for yawn and control videos, and proportions for male- and female- yawn videos. A Linear Mixed Model (LMM) was used to assess the effect of the group’s grooming distribution, as a proxy for relationship quality, on the proportion of yawn-videos of each individual to which each individual responded with at least one yawn, while controlling for subject- and model identity (random effects), and also including subject- and model sex, and proximity [12] as fixed effects into the model.

## Results

**Figure 1:**
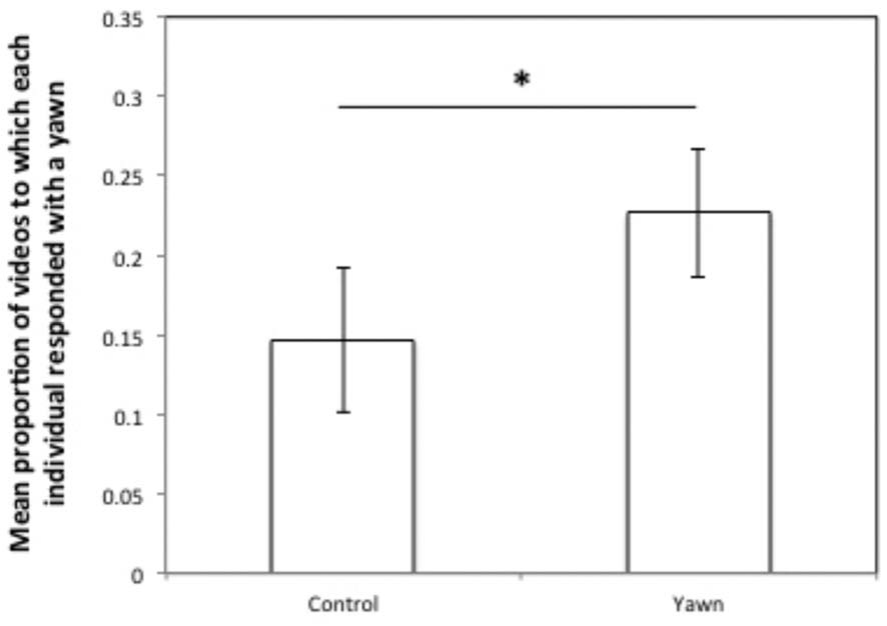
Yawn Contagion. Mean proportion of videos (control vs. yawn) to which each individual responded with a yawn, *p = 0.019.

As predicted, per individual the proportion of yawn-videos to which it responded with at least one yawn was significantly higher than the proportion of control-videos to which it responded (Wilcoxon: *T^+^* = 69, *n* = 15, *p* = 0.019; Figure 1). Yawn contagion was similar for both subject sexes, since there was no significant effect of the sex of the subject on the proportion of yawn-videos to which it responded (Mann-Witney test, *U* = 13.5, *n = *15, *p = *0.515).

**Figure 2:**
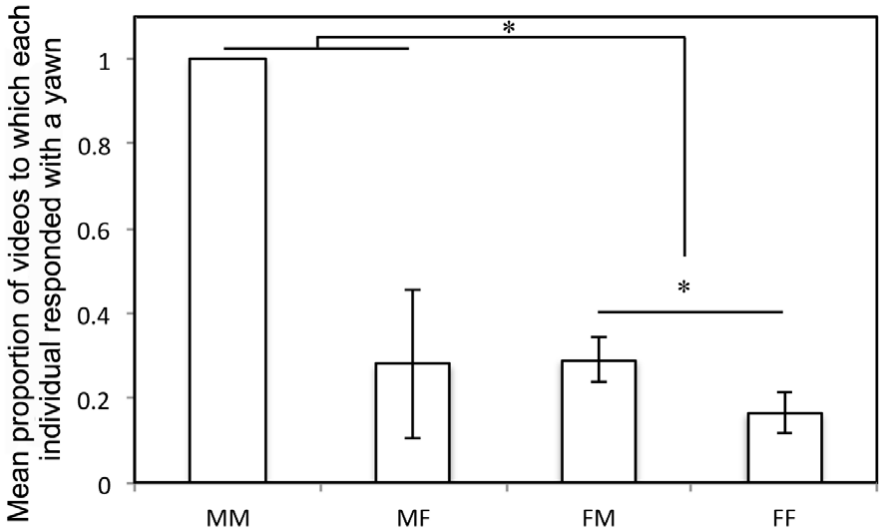
Yawn Contagion in relation to subjects’- and models’ sex. Mean proportion of videos to which each individual responded with a yawn: males’ **(n = 3)** reaction to male yawn videos (MM), males’ reaction to female yawn videos (MF), females’ **(n = 11)** reaction to male videos (FM), and females’ reaction to female videos (FF). *p>0.05.

However, the sex of the model had a significant effect on yawn contagion, i.e. the proportion of male yawn-videos to which each individual responded was significantly larger than the proportion of female yawn-videos to which each individual responded (Wilcoxon: *T^+^* = 71, *n* = 13 *p* = 0.012). This effect was larger for males than for females (two-way ANOVA, *interaction subject- and model sex*, *F* = 12.93, *df. = *1, *p = *0.001; Figure 2), yet also present in females (Wilcoxon: *T^+^* = 48, *n* = 11 *p* = 0.037).

We found no effect of the grooming distribution on the proportion of yawn-videos of each individual to which each individual responded (LMM: *F* = 1.796, *numerator df* = 1, *denominator df* = 140.072, *p* = 0.182). However, largely similar to the above mentioned ANOVA, the LMM did reveal a significant effect of both subject sex (*F* = 4.081, *numerator df* = 1, *denominator df* = 50.979, *p* = 0.049) and model sex (*F* = 5.906, *numerator df* = 1, *denominator df* = 62.304, *p* = 0.018), and a slight, yet non-significant effect of the interaction between both variables (subject sex * model sex: *F* = 3.054, *numerator df* = 1, *denominator df* = 139.695, *p* = 0.083).

## Discussion

Our study confirms previous studies [10], [11], [18], and shows that yawning is contagious for chimpanzees. This, however, was the first study that used an experimental group set-up to investigate yawn contagion. In contrast to studies that voluntarily separate animals, this group set-up allowed us to test *all* animals in our study population, and also avoided any negative effects of the stress of separation, a possible confound in yawning studies [13], [22]. A group set-up, however, makes the analyses of yawning contagion more challenging, since it is hard to distinguish who causes the yawning contagion; either the actual model or a conspecific that previously responded to the model with a yawn.

In contrast to other studies [4], [12], our study did not show an effect of relationship quality on contagious yawning. The lack of an effect of relationship quality on yawn contagion in our group of chimpanzees may be a result of possible small variation in relationship quality due to the relatively small size of the group in comparison to natural groups [24], or due to the long and stable residence of the members of our study group. Alternatively, differences in outcomes may result from different methods; whereas the studies on humans [4] and gelada baboons [12] rely on observed spontaneous yawns, our study employed an experimental approach and stimuli may be somehow artificial. Nonetheless, the supposed link between contagious yawning and relationship quality follows from the prediction that empathy may be the proximate mechanisms driving the contagiousness of yawning [1]. However, as our data do not support a link between yawn contagion and factors indicating strong social bond, the proposed relationship between yawn contagion and empathy [1–4, 12, 15, 18, *but see* 8] is not supported by our findings. Altogether, the relationship between empathy, relationship quality and yawn contagion in animals needs to be further investigated.

This study does show strong sex effects of both the model and the subject on the contagiousness of yawning. We show that male yawns are far more contagious than those of females, and that this effect is most apparent when the subject is a male too. From an ultimate perspective, these results follow the prediction that yawning contagion functions to facilitate the synchronisation of behaviour [22], one would then predict that yawning is only contagious if the model’s behaviour is relevant to others. In chimpanzees the higher contagion of male than female yawns is consistent with males being the dominant sex [24] and initiating group movement [25], [26]. In addition, individuals of the bonded sex (males [29]) are also expected to influence each other more, and this is consistent with the strong effect males have on other males. Similar patterns have been found among female-bonded gelada baboons, where yawn contagion was most apparent among females [12]. Moreover, this line of thought is bolstered by recent findings that the yawns of ingroup members are far more contagious than those of outgroup individuals [18], since signals of outgroup individuals, although outgroup members and their signals are very interesting [18], have little relevance with regard to the synchronization of group behaviour. Also, these results are in line with recent findings showing that for dogs the yawns of familiar caretakers are more contagious than those of unfamiliar humans [15], since the signals of caretakers are for more salient for dogs with regard to the synchronisation of behaviour than those of unfamiliar humans. And, although not supported by our findings, also the correlations between measures of social bonds and yawn contagion [4], [12] are consistent with this hypothesis, since signals of socially close individuals will be more relevant than those of less close individuals. Nonetheless, our data do not reveal whether yawn contagion actually leads to the synchronisation of behaviour, and consequently, studies that truly explore the ultimate function of yawn contagion are still needed.
